# Perspective: a systems approach to diabetes research

**DOI:** 10.3389/fgene.2013.00205

**Published:** 2013-10-16

**Authors:** Martin Kussmann, Melissa J. Morine, Jörg Hager, Bernhard Sonderegger, Jim Kaput

**Affiliations:** ^1^Nestlé Institute of Health Sciences SA Lausanne, Switzerland; ^2^Faculty of Life Sciences, Ecole Polytechnique Fédérale Lausanne, Switzerland; ^3^Faculty of Science, Aarhus University Aarhus, Denmark; ^4^The Microsoft Research - University of Trento Centre for Computational and Systems Biology Rovereto, Italy; ^5^Department of Mathematics, University of Trento Trento, Italy; ^6^Endocrinology, Diabetology and Metabolism Division, Centre Hospitalier Universitaire Vaudois, University of Lausanne Lausanne, Switzerland

**Keywords:** type 2 diabetes, nutrition, prevention, systems biology, genomics, proteomics, metabonomics

## Abstract

We review here the status of human type 2 diabetes studies from a genetic, epidemiological, and clinical (intervention) perspective. Most studies limit analyses to one or a few omic technologies providing data of components of physiological processes. Since all chronic diseases are multifactorial and arise from complex interactions between genetic makeup and environment, type 2 diabetes mellitus (T2DM) is a collection of sub-phenotypes resulting in high fasting glucose. The underlying gene–environment interactions that produce these classes of T2DM are imperfectly characterized. Based on assessments of the complexity of T2DM, we propose a systems biology approach to advance the understanding of origin, onset, development, prevention, and treatment of this complex disease. This systems-based strategy is based on new study design principles and the integrated application of omics technologies: we pursue longitudinal studies in which each subject is analyzed at both homeostasis and after (healthy and safe) challenges. Each enrolled subject functions thereby as their own case and control and this design avoids assigning the subjects *a priori* to case and control groups based on limited phenotyping. Analyses at different time points along this longitudinal investigation are performed with a comprehensive set of omics platforms. These data sets are generated in a biological context, rather than biochemical compound class-driven manner, which we term “systems omics.”

## INTRODUCTION

Type 2 diabetes mellitus (T2DM) is a complex disease with epidemic proportions. The International Diabetes Federation estimates a total of 371 million type 2 diabetics worldwide^1^. China and India alone account for more than 150 million cases. Hence, there is a public health, economic, scientific, and ethical call for a proactive and preventive approach to the individual and public health burden caused by diabetes and its co-morbidities. Scientifically based preventive approaches should complement the reactive, pharmaceutical approach of management and treatment.

Type 2 diabetes mellitus is a multi-factorial condition that can already occur during gestation and has variable onset, severity, and outcome in juveniles, adults, and the elderly. Genetic (predisposition), epigenetic (developmental programing), and environmental factors (diet and physical activity) contribute to T2DM, with epigenetic contributors so far only being suggested from an epidemiological and animal model perspective. The complexity of the T2DM phenotype has challenged the fragmented scientific approaches, typically focusing on either genetic, or environmental (diet, lifestyle), or socio-economic conditions in isolation rather than on multi-scale, longitudinal, systems-level studies, which we explain here.

Although systems biology is considered an emerging paradigm for biological research, its roots can be traced back to von Bertalanffy (1951). Systems research typically refers to integrating combinations of high-dimensional (epi)genomic, transcriptomic, proteomic, or metabonomic data. Conceptual experimental approaches have been proposed for obesity, diabetes, and cardiovascular disease (MacLellan et al., 2012; Zhao et al., 2012; Meng et al., 2013). However, only a few multi-scale experimental results have been reported (Morine et al., 2010, 2011; Lau et al., 2012). Many of these examples analyze the physiological system – that is, the system is defined as processes occurring within the body. However, environmental factors such as nutrition, activity, rest/sleep, exposures to toxins and stress, and psychological factors, are known to influence an organism’ s physiology, too.

**FIGURE 1:**
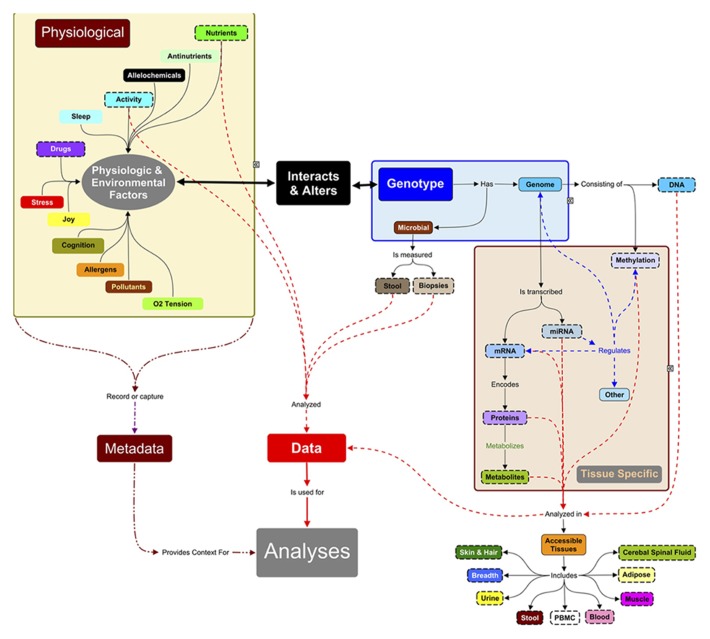
**Systems approach to analyses of complex traits.** Systems research typically focuses on data measured (objects with dashed lines) from accessible tissues (adipose, muscle, occasionally other biopsies) and body fluids. Hence, many systems studies are analyzing a small subset of, e.g., the total metabolite complement. Since the measured molecules (proteins, RNAs, metabolites, etc.) are influenced by the expression of genomic information, DNA must also be characterized: the level and relationships between biomarkers are context-specific and the context here is the genes inherited from parents. DNA methylation, microRNAs, and chromatin modifying enzymes also change the expression of genetic information and can be measured in some tissues. These regulators are in turn regulated by environmental signals of nutrients and bioactives in food, and by a large number of physiological factors (sleep, ability to handle stress, joy) and personal (activity) and public environmental conditions (pollutants, allergens, alleochemicals from food, etc.). Many of these factors are not measured in systems research but they directly or indirectly can influence expression of genetic information. These metadata need to be captured in future experiments to understand the context of the physiological measurements and outcomes. The challenge of systems research is integrating these multi-scale data sets, which is still in its infancy.

Our definition of a “multi-scale systems” approach means deep characterization of subjects at omic and clinical level, and – importantly – as many environmental factors as possible, and integration of these data (**Figure 1**) in interaction networks (including both functional interactions and statistical correlations). “Longitudinal” means that every study subject is assessed multiple times and ideally with challenges to homeostasis (van Ommen et al., 2009). These combined approaches differ from the standard methodology, in which single homeostatic “snapshots” are taken between *a priori* defined groups of subjects, which are assumed to be matched for age, gender, genetic, socio-economic, or other confounders. These studies are furthermore often technology-driven, assuming that improvements in high-throughput genomics, transcriptomics, proteomics, or metabonomics suffice to correlate molecular signatures with disease phenotype. This goal has, however, been difficult to achieve, particularly in complex diseases, due in part to molecular and clinical heterogeneity between “cases.”

We do not conduct our human clinical studies by assigning subjects to groups based on a limited number of parameters. Rather, we propose a longitudinal study design, in which the same study subject is repeatedly monitored over time and individuals with like response are assigned to metabolic response groups only after multidimensional characterization. This approach uses each subject as their own control, challenging the classical case–control study design. Such an individual-focused longitudinal approach centers on analysis of trajectories of each measured variable over time.

Moreover, instead of studying a phenotype at homeostasis only, we expose subjects to metabolic challenges (e.g., oral glucose tolerance, high-fat meals or diets, physical exercise, or cognitive tests) and probe their metabolic elasticity, i.e., how rapidly or slowly they return to homeostasis after such safe, acute challenges. These repeated challenges and their omic read-outs of the body’ s reactions can enable better definition of an individual’ s health status and, potentially, their susceptibility to disease. A case in point: women who develop gestational diabetes during the stress of pregnancy are at a five-fold greater risk for developing T2DM; hence, metabolic responses to homeostatic challenges in healthy subjects may identify susceptibilities to later disease, which would derail their healthy trajectory before symptoms occur. These insights can in turn reveal early biomarkers for emerging metabolic disease and signatures for metabolic groups.

The integration of the comprehensive analyses of an individual’ s genetic predisposition, epigenetic programing, and reaction at all omic levels may pose problems of too high dimensionality. However, we take a systems approach rather than a biochemical compound-class approach in the sense of completely mapping out key pathways and networks in diabetes (i.e., identifying and quantifying all enzymes, nutrients, and metabolites in those pathways). We term this “systems omics” as opposed to “classical” proteomics/metabonomics/lipidomics.

## HUMAN (EPI)GENETIC INDIVIDUALITY WITH REGARD TO T2DM

Many genetics-oriented studies of diabetes have not taken into account any information on an individual’ s environment, such as nutrition, physical activity, or lifestyle: typically, genome-wide association studies (GWAS) in cohorts derived from diabetic and healthy populations have identified multiple genetic, population-attributable (fraction) risk loci. These markers are usually single-nucleotide polymorphisms (SNPs), which – in combination – were expected to predict disease risk in individuals. However, they explain only a fraction of the disease risk, partly because of the misperceived definition of “population-attributable fraction” (PAF), which is the decrease in disease incidence if the allele were not present in that population (Rockhill et al., 1998; Kaput, 2008). Since GWAS is determined by the average occurrence of a SNP between cases and controls, the risk allele is actually a PAF and not the personal or individual risk factor. In fact, family history is a better predictor of diabetes risk than SNPs identified by GWAS (Meigs et al., 2008).

A number of authors have identified limitations in the GWAS designs (McCarthy et al., 2008; Goldstein, 2009; Manolio et al., 2009; Need and Goldstein, 2010):

– Poorly defined phenotypes.
– Indirect scoring of causal SNPs by distant gene markers.
– Challenge to find rare alleles.
– Insufficient consideration of gene × gene interactions (epistasis).
– Insufficient consideration of gene × environment interactions.
– Copy number variants.
– Epigenetic factors.

In addition, human populations are not at equilibrium with exponential population growth occurring only during the last 150 years. The consequence of this rapid expansion is that more recent and rare variants exist than assumed by the common variant/common disease model, which is the theoretical basis of the GWAS experimental designs (Cargill and Daley, 2000; Hirschhorn and Daly, 2005; Iyengar and Elston, 2007; Fu et al., 2013). Hence, the sample size for a representative proportion of the population roughly equals the effective population size.

Gene–diet interactions have been analyzed in humans, but typically in a single-gene versus single-nutrient design, where a complex phenotype (such as response to nutrient) is thought to be caused by a single variant in one gene out of a total of ~20,000 human genes. Although selected gene–diet associations have been replicated in multiple populations, e.g., in the study “Association between the APOA2 promoter polymorphism and body weight in Mediterranean and Asian populations: replication of a gene-saturated fat interaction” (Corella et al., 2011), the effect size of these associations is known to explain only a small percentage of the overall phenotype. Alternatively, classical nutritional epidemiology studies associating a nutrient to a phenotype were performed in clinical cohorts or in entire (sub)populations, and these studies neglected the genetic contributions to the phenotype. This design is based on the assumption of finding the statistical main effect of the diet or lifestyle.

It is well established that genetic factors contribute significantly to T2DM. Estimates of heritability for T2D range from 50% to 80% and are much higher than for T1D. However, these estimates rely on the assumption that environmental exposures are equal for those that are affected and those that are not. This may not be true, even when comparing monozygotic to dizygotic twins (Loos et al., 2001; Ollikainen et al., 2010). Studies of maturity onset diabetes of the young (MODY), a highly penetrant monogenetic form of type 2 diabetes, have proven that single gene mutations can lead to T2DM (Johansson et al., 2012; Mitchell, 2012), although the age of onset, severity, outcome, and age of death demonstrate the importance of modifying genes. Many SNPs affect gene expression or protein function more subtly than loss-of-function mutations. While not explaining most of the estimated heritability, GWAS have provided new insights into the etiology of T2DM. One striking and unexpected outcome of genetic studies of both MODY and late onset T2DM is that most genes identified so far are involved in insulin secretion. A caveat though is that over 90% of GWAS were conducted in Europeans and the findings cannot be generalized to other populations. Myles et al., for example, showed that allele frequencies of a subset of 25 GWAS-identified risk alleles were not consistent with contributions to disease incidence in Africans or Asians (Myles et al., 2008). Others have shown that different pathways that alter glucose levels (i.e., insulin sensitivity, gluconeogenesis, insulin secretion) differ between ancestral groups (Misra and Vikram, 2004). This is important from a systems perspective, as it suggests that a given phenotype can arise from distinct perturbations of system function, and thus puts into question the assumption of many GWA studies that all cases are identical from a patho-physiological viewpoint.

Nevertheless, the fact that so far most genes identified by GWAS are involved in insulin secretion may shed new light on the role of obesity and insulin resistance in T2DM etiology. Indeed, one may speculate that T2DM (like type 1) is fundamentally a β-cell disease and obesity-related insulin resistance is a secondary risk factor, which may push “at-risk” individuals across the disease threshold. The knowledge gap is whether genetic variants involved in insulin secretion independently increase the risk of T2DM in addition to the physiological effects caused by high body mass index (BMI).

If we want to prevent T2DM, we need to disentangle the causal variations and environmental factors. To do this we need new approaches to studying genetic variation that do not rely on a design, in which type 2 diabetics with different causal variants are compared to controls, because individuals classified as T2DM and control have highly variable phenotypes. The future and the success of genetic studies of T2DM will not be solved by ever bigger association studies, but rather in well-defined studies enrolling carefully phenotyped subjects, who should be exposed to a (dietary or other) external challenge to reveal their individual responses. New sequencing technologies are expected to identify additional rare variants with somewhat higher effect sizes. In addition to this revised study design, data acquisition, processing, and interpretation also need to be revisited: all molecular and clinical data need to be regarded as a single input into a systems approach to eventually unravel those mechanisms that are the most important risk factors for a given group of individuals. Only then can we design effective interventions, either nutritional, life-style or drug-based, to prevent or at least delay the development of this disease.

## HUMAN STUDIES ON T2DM – TODAY AND TOMORROW

Many, if not most, human studies have been designed in a case – control design with the most important consideration being the power of the experiment to detect the null hypothesis (Bader, 2001; Longmate, 2001; Lee and Whitmore, 2002; Teo, 2008; Visscher et al., 2008). An unintended consequence has been an increasing misclassification: adding subjects to case or control groups based on simplistic phenotypic characterization. For example, most population-based studies use fasting plasma glucose levels or other homeostatic measures associated with T2DM. However, elevated fasting glucose levels could be produced by decreased insulin production, increased gluconeogenesis, insulin insensitivity, or increased glucose uptake in the intestine (Kaput et al., 2007; Malecki and Skupien, 2008). Individuals differ in these pathways and may have different genetic variants that contribute to these glucose abnormalities. Grouping all subtypes of T2D therefore decreases the signal and increases noise in these case–control analyses. The failure to account for environment–gene interactions also confounded GWAS analyses.

Two related approaches may improve study designs and both require more comprehensive phenotyping before analyzing genetic differences. The first approach is to compare genetic makeup of individuals at the extremes of a given phenotype (Ahituv et al., 2007; Kaput, 2008; Khor and Goh, 2010). This strategy may work for highly differentiated phenotypes, for example, tall versus short stature (Lettre, 2009) or the subset of metabolic phenotypes that can be clearly discriminated by homeostatic measurements. The second approach uses short-term (acute) or long-term (chronic) challenges to homeostatic systems or, ideally, a combination of them. The oral glucose tolerance test is used for assessing glucose regulation and a prime example of such acute challenges. Other nutritional and functional challenges (e.g., exercise) may also be used for assessing metabolic robustness (van Ommen et al., 2009). Results from such challenge studies using extensive omics, similar to the approach described in this article, have been published (Wopereis et al., 2009; Heinzmann et al., 2012) and confirm the large intra-individual variability observed at homeostasis. However, small sample sizes limited the ability to cluster individuals with similar responses. O’ Sullivan and coworkers, for example, could group individuals based on biomarker patterns following long-term vitamin D supplementation (O’ Sullivan et al., 2011).

We propose here to combine acute challenges of homeostasis, such as oral glucose tolerance, with longitudinal analyses, ideally following defined nutritional or activity interventions which alter physical condition, such as weight loss or improved cardiovascular health. The response to single homeostatic challenges is influenced by the current physiology of the individual: to illustrate, O_2_ consumption measured at homeostasis or in acute challenges (e.g., upon a single exercise bout) will likely differ before and after months of athletic training. So, which time point and physiology accurately measures an individual’ s aerobic capacity robustness?

The study design and execution suggested here, including extensive omics at homeostasis and after acute challenges, measured longitudinally, increases both cost and complexity of assessing health and disease. The current model of funding and conducting biomedical research was designed in the pre-genomic era. The average research grant in the USA was US$ 482,276 in 2012 and most grants are 3 year awards, with resources too small to conduct multi-scale scientific research^2^. While smaller scale science funding must be maintained, the complexity of biological processes requires complex analysis that can only be done through extensive collaborations in consortia. Many examples of these consortia now exist: the Human Genome (Lander et al., 2001), the International HapMap (International HapMap Consortium, 2003), the ENCODE (Birney et al., 2007), and the 1000Genomes (The 1000 Genomes Project Consortium) projects (organized in the United States); and many of the Framework projects in Europe such as NuGO^3^, Food4Me^4^. The paradigm of funding and conducting biomedical research is changing.

Human studies of systemic, metabolic conditions like T2DM must not be restricted to the assessment of the human host only: humans carry ~100 times more bacterial than human cells in and on their body, colonizing mucosal surfaces (e.g., gut, oral cavity, vagina) and the skin (Ursell et al., 2012). The intestinal microbiota are the most complex of these bacterial communities (Dimitrov, 2011) and centrally involved in host metabolic (Harris et al., 2012) and immune health (Hakansson and Molin, 2011). We therefore proposed in an earlier perspective published in this journal an “extended nutrigenomics” approach encompassing the host, microbial and food genomes to better understand gene–diet interactions in humans (Kussmann and Van Bladeren, 2011). Today’ s insights into host–microbiome relationships derive from large-scale (earlier 16 sRNA sequencing, nowadays deep sequencing) studies and mainly reflect associations between a microbial population census on the one hand and a host condition on the other hand. It has for example been shown that human subjects with different metabolic conditions (e.g., diabetes, Larsen et al., 2010 or obesity, Turnbaugh et al., 2006) have different gut microbiota. Next steps have recently been taken toward establishing a more causal (Knaapen et al., 2013) than purely associative relationship (Cox et al., 2013): the biochemical activities of such gut microbial populations have been probed by metabonomic means and so-termed “core” human microbiomes have been characterized with metabolic functions generalizable between individuals (Huse et al., 2012; Li et al., 2013). This may pave the way for tailoring diets to metabolic groups of consumers and patients to induce or maintain a favorable microbiome (Nicholson et al., 2005; Wu et al., 2011). Possible causal relationships between gut microbes and host health need to be considered from both perspectives: microbes possibly altering health phenotype and host factors possibly determining microbial composition. Yokota et al. (2012) took it from the host end asking whether bile acid is a determinant of the gut microbiota on a high-fat diet and discussing how the identification of host factors determining the gut microbiota can contribute to understanding the causality between changes in gut microbiota and disease development. Vrieze et al. (2013) even took the ultimate step from causality to possible treatment considering fecal transplantation as a clinical therapy for restoring intestinal microbial balance in human disease.

## OMICS MONITORING OF HUMAN STUDIES ON T2DM – TODAY AND TOMORROW

Omics studies in nutrition and diabetes have typically been performed in a technology-*driven*, rather than a technology-*rooted* manner. While the advent of high-throughput and comprehensive genomics and other omic platforms have advanced biological knowledge, the emphasis on single technology-driven nutritional and biomedical research projects still limits assessing health and disease conditions and trajectories: there are a number of investigations that attempt to find either genetic (Billings and Florez, 2010), or epigenetic (Pinney and Simmons, 2010), or proteomic (Rao et al., 2009; Riaz et al., 2010a,b) or metabonomic (Bao et al., 2009; Huo et al., 2009; Griffin et al., 2011) biomarkers for T2DM. Our suggestion is that cross-platform approaches are needed to truly interrogate physiological processes.

While genetic markers can inform on inborn predisposition and susceptibility, epigenomics can reveal those genetic marks altered by the environment, particularly diet (Waterland and Jirtle, 2003, 2004; Gluckman et al., 2009; Low et al., 2011). Epigenetic modifications increasingly appear to deliver the molecular basis for the intuitive observation that environment shapes phenotype during lifespan and even across generations (Morgan and Whitelaw, 2008; Morris, 2009). However, epigenetic marks do not only affect DNA bases (methylation) but also the “DNA-packaging” proteins such as histones (Jenuwein and Allis, 2001), other chromosomal components that regulate transcription, and small RNAs (Bei et al., 2007; Choudhuri, 2011). Despite recent developments of proteomics in deciphering the histone code (Trelle and Jensen, 2007; Sidoli et al., 2012) and the epigenetic interplay between DNA- and histone modifications, there are few studies that integrate proteomics and DNA sequencing to generate a more complete picture of epigenetic marks that affect gene expression and phenotype after environmental exposure (Fuks et al., 2003; Johnson et al., 2007).

A body of pure proteomic approaches is emerging that analyzes energy metabolism-relevant tissues across different conditions (pancreas, Chen et al., 2007a, b; Kim et al., 2008; muscle, Wang et al., 2010; Giebelstein et al., 2012; adipose tissue, Ahmed et al., 2010; Perez-Perez et al., 2012; pancreatic beta cells, Song et al., 2009; Maris et al., 2011; and mitochondria, Forner et al., 2006; Chen et al., 2012). These tissues were typically isolated from normal (control) and diabetic (case) human subjects, mouse strains or genetic mouse models. Potential protein/peptide biomarker signatures are being associated by analyzing blood or urine, in order to facilitate less invasive sampling.

Metabonomic studies in T2DM can be classified in those based on (proton) nuclear magnetic resonance (^1^H-NMR) spectroscopy (Rezzi et al., 2007; Lindon and Nicholson, 2008; Collino et al., 2009), and those deploying mass spectrometry (MS; Toyo’ oka, 2008; Kertesz et al., 2009; Mishur and Rea, 2012). NMR-enabled metabonomic profiling in the context of diabetes was typically done on human urine (Kussmann et al., 2006; Faber et al., 2007; Maher et al., 2009; Zhao et al., 2010). The intrinsic NMR advantages like high-throughput, robustness, and minimal sample preparation are now being complemented by the higher sensitivity and greater structure elucidation power of MS (Lenz et al., 2005; Kussmann et al., 2008). However, investigations integrating metabonomics with proteomics or even genetics or epigenetics have remained the exceptions (Holmes et al., 2001; Vilasi et al., 2007; Hardiman, 2011).

Current proteomic discovery platforms typically span a dynamic range of 10^4^ to 10^5^ whereas the protein concentration range in human blood plasma is estimated to be 10^12^ (Lescuyer et al., 2007; Surinova et al., 2011). Similarly, current metabonomic workflows cover a range of lipophilic and hydrophilic metabolites (Whiley et al., 2012); carbohydrates (Soo and Hui, 2010), amino acids and ketones (Suhre et al., 2010); and the complexity of lipid biochemistry has resulted in lipidomics as a specialized new omics discipline (Ejsing et al., 2009; Shevchenko and Simons, 2010). These proteomic and metabonomic studies are typically designed from a biochemical, compound-class perspective.

Complementary to the aforementioned study designs we propose here “systems omics,” i.e., to join forces of transcriptomics, proteomics, metabonomics, and lipidomics to completely map out – that is identify and quantify all (micro-)nutrients, metabolic enzymes and their substrates and products – in a given biochemical pathway. We thereby aim at deeply characterizing the dynamics of those pathways that have emerged as consistently (de-)regulated in a specific health condition and under relevant environmental – such as dietary – influences.

The value of a systems approach was recently demonstrated by Jain et al. (2013), who described the molecular architecture of T2DM pathophysiology using multi-omics data, combined with physical and genetic interaction networks. Their work was largely motivated by the observation that, despite the symptomatic complexity of T2DM (incl. alteration of immune function, oxidative stress, and nutrient metabolism), the majority of GWAS to date have failed to highlight these processes as genetic determinants of T2DM onset/progression. Starting with publically available T2DM GWA data, the authors demonstrated that the only significantly enriched pathway with T2DM SNPs was that of MODY^5^. By extending these T2DM SNPs to their first-degree neighbors in physical and genetic interaction networks (creating a “T2DM interactome”), and incorporating gene expression data from multiple tissues in healthy and diabetic individuals, they identified a range of pathways with known relevance to T2DM. In particular, the TGFβ signaling pathway contained multiple genes present in the GWA, interactome, and transcriptomic data sets. Jain et al. (2013) showed that a multi-omics approach can reveal disease-related processes that may not be evident in a single omics data set, and can also aid biological interpretation of GWA data.

Based on the components identified in key T2DM-related pathways, we propose that MS assays for all nutrients, enzymes, and metabolites in these contexts be developed and applied. These assays are based on single- and multiple reaction monitoring (SRM and MRM) of target molecules, so called MS-based enzyme-linked immunosorbent assays (ELISAs; Maiolica et al., 2012), which use sequencing of specific peptides that are unique surrogates for their parent protein. For measuring metabolites in this “systems omics” strategy, intact mass-based metabolite identification and quantification (the latter requiring either isotope-labeled or unlabeled internal standards that are either chemically identical or highly similar to the target analytes) is of high interest. Concomitantly, high-resolution and high-mass accuracy MS have become the preferred technology platforms for global, quantitative metabolite profiling. Targeted SRM and MRM methods that can filter, detect, identify, and quantify selected metabolites against complex biochemical matrices are powerful platforms in clinical metabonomics, particularly in systems omics studies proposed here.

Omics integration is one way to address the spatial and temporal complexity of type 2 diabetes, being a multi-organ disease with multiple and interrelated contributing dysfunctions, rather than “just” a beta cell disorder. A systemic, low-grade, chronic inflammation drives metabolic deteriorations in several organs in T2DM. This raises the question of both meaningful and feasible sampling of body tissues and fluids and how one could extrapolate from intra-cellular networks to a systemic multi-organ disease. Repeated sampling of tissue biopsies during a longitudinal study is not feasible, because key organs for metabolic function like pancreas, liver or gut are only accessible by highly invasive means. We are therefore restricted to the periphery for sampling, i.e., blood, urine, and stool. Blood and urine omics have become standard approaches to assess protein, peptide (Crosley et al., 2009), lipid (Song et al., 2013), and metabolite (Guy et al., 2008) profiles. These profiles become more meaningful when sampled in the same individual over time and when metabolic/physical challenges are compared to the resting condition, as we propose here. Moreover, biomolecular blood profiles can inform about homeostasis (or deviation thereof) whereas urine profiles can indicate the body’ s measures (e.g., secretion) to achieve or restore this homeostasis. White and red blood cells have unique, cell-specific metabolisms and may inform about metabolic or immune status if analyzed in conjunction with hematological analysis of cell types (platelets, eosinophils, other white blood cell types; Crosley et al., 2009). Stool samples have become the non-invasive choice to access the gut microbiota (see previous section; Flores et al., 2012a,b). Hence, only repeated, peripheral, non-invasive sampling over time and across challenges is both meaningful and feasible to generate systems-level insights into a multi-organ disease like T2DM in humans.

## RECONSTRUCTING THE SYSTEM OF T2DM WITH NETWORK ANALYSIS

With exponential reduction in cost of data generation, processing and managing, it has become more common to measure multiple types of omics data in a given study. However, translation of these omics data sets into concrete knowledge of biological systems is contingent on informative methods for multi-omics analysis. The simplest approach to analysis of, for instance, a transcriptomic and proteomic data set, would be to generate lists of genes and proteins that respond to a given perturbation or correlate with a given phenotype, then attempt to build a biological interpretation *post hoc*. However, this analytical approach does not capture the functional interactions within and between the different classes of molecular species, which ultimately coalesce to form the biological system of interest.

Biological network analysis offers a natural framework for analysis and interpretation of omics data sets. This said, pathway analysis is currently more widely adopted than network analysis, owing in part to strong methodological development and implementation, as well as straightforward biological interpretability. The limitation of such pathway analysis is that the pathway models, by design, do not capture the intersection of these pathways that create the entire system. While global interaction networks inherently capture these intersections, analysis of these networks is uniquely challenging due to their considerable size. The I2D database^6^, for example, contains 173,338 human protein interactions as of February 2013. A practical consideration in the analysis of such data sets is the presence of false-positive – which are pervasive in online interaction databases^7^ – or otherwise irrelevant interactions. Therefore, utilization of a global interaction data set in the context of an omic analysis often requires manual curation to remove, possibly, interactions inferred by homology, or interactions that do not physically occur in a tissue of interest. Once such a framework network is identified, the next challenge is to identify, which sub-regions (i.e., collections of nodes) significantly respond to a given perturbation, or correlate with a given phenotype. A range of algorithms have been developed for this purpose, including heuristic (Liu et al., 2007) and exact (Qiu et al., 2009) community detection approaches, as well as network path tracing (Morine et al., 2011).

Similar to (and possibly driven by) the to-date trend of generating and analyzing a single omic data type, molecular interaction networks (as stored in interaction databases such as MetaCyc, Bind, I2D, IntAct, etc.) commonly represent a single type of interaction – for instance, metabolic, protein-protein, or regulatory interaction. However, it is intuitive that these classes of interactions do not occur in isolation: metabolic reactions produce metabolites, which may regulate signaling cascades, which in turn may activate transcription regulation. Reconstruction, curation, and analysis of such multi-level networks will be instrumental in understanding complex, multi-factorial diseases such as T2DM from a systems standpoint.

**FIGURE 2:**
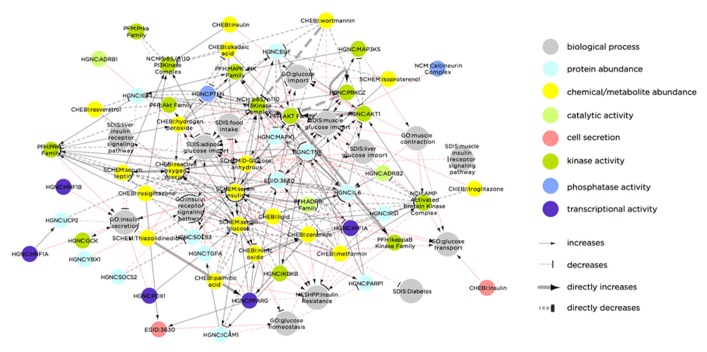
**Biological Expression Language framework network of biological molecules that are causally linked to the T2DM molecular phenotype.** Node color indicates molecule type and/or molecular process. Red links indicate molecular interactions, while gray links indicate process-molecule causal relationships. The network was constructed by extracting all nodes in the BEL knowledgebase containing the terms “insulin secretion,” “insulin receptor signaling,” “glucose transport,” “glucose import,” or “glucose homeostasis,” and extending to the first degree neighbors of these seed nodes. The result is a network containing both molecular nodes (e.g., PRKCZ) and biological process nodes (e.g., liver glucose import), as well as the causal relationships between them. Some molecular nodes in the network additionally contain a dynamic property, indicated in the node color. For instance, cell secretion (as indicated in red) of “CHEBI: Insulin” increases “GO: glucose transport.” Similarly, transcriptional activity (purple) of “HGNC: PPARG” increases “GO: Insulin receptor signaling pathway.” Abbreviations in the network nodes are as follows: CHEBI, Chemicals of Biological Interest names (); EGID, Entrez Gene IDs (); GO, Gene Ontology names (); HGNC, Human Genome Nomenclature Committee (); MESHD, Medical Subject Heading Disease names (); MESHCL, Medical Subject Heading Cellular Structure (); MGI, Mouse Genome Information gene symbols (); NCH, Human Molecular Complex names (); PFH, Human Protein Family names (); PFM, Mouse Protein Family names (); RGD, Rat Genome Database gene symbols (); SPAC, SwissProt accession numbers ().

A notable example of this type of multi-level network is the Biological Expression Language (BEL) framework^8^, which includes a knowledge base of curated, directed interactions and a dedicated language (i.e., a syntax and semantics). The BEL framework contains information-rich interactions within and between different classes of biological components. Each interaction is categorized by type (e.g., binding, reaction, translocation, etc.) and includes a line of “evidence” text parsed from the associated publication, describing the context of the interaction. As an example, **Figure 2** illustrates the BEL network using a list of T2DM-related molecular processes (based on a node keyword search for “insulin secretion,” “insulin receptor signaling,” “glucose transport,” “glucose import,” or “glucose homeostasis”) as seed nodes, and then extending to the first-degree neighbors. Apart from demonstrating the complexity of interactions related to T2DM, this sub-network illustrates the range of biological molecule types that are causally associated with the diabetic phenotype.

Knowledge bases such as BEL demand a great deal of effort to be curated and would therefore benefit from more scientific community contributions in terms of reporting identified molecular interactions. Public transcriptomic databases have benefitted considerably by the common editorial requirement for deposition of transcriptomic data into databases such as Gene Expression Omnibus (GEO) and ArrayExpress. A similar requirement for deposition of interaction data in a standardized (and information-rich) format before publication of a manuscript could be a way of strengthening these interaction databases, which would in turn enhance biological systems analysis and understanding.

## CONCLUSION: A SYSTEMS APPROACH TO T2DM RESEARCH

We suggest here an integrated systems approach of longitudinal multi-omics challenge studies to reveal early molecular signs of diabetes. We argue that this strategy will identify better biomarkers and diagnostics that would in turn enable personalized intervention through tailored diets prior to onset of chronic disease. These interventions would consist of (micro)nutrients and other functional ingredients that would be used for disease prevention rather than disease management or cure. Having defined this as one of the key objectives in diabetes research, we acknowledge at the same time the challenge of completely mapping out a complex human tissue or body fluid at proteomic or metabonomic level.

## Conflict of Interest Statement

The authors declare that the research was conducted in the absence of any commercial or financial relationships that could be construed as a potential conflict of interest.

## Footnotes

1 http://www.idf.org/diabetesatlas/5e/Update2012

2

3

4

5

6 http://ophid.utoronto.ca

7

8

## Abbreviations

LC: liquid chromatography
MODY: maturity onset diabetes in the young
MS: mass spectrometry
T2DM: type 2 diabetes mellitus.
